# Reasons for patient non-compliance with compression stockings as a treatment for varicose veins in the lower limbs: A qualitative study

**DOI:** 10.1371/journal.pone.0231218

**Published:** 2020-04-28

**Authors:** Jian-Mei Gong, Jian-Shi Du, Dong-Mei Han, Xin-Yu Wang, Shao-Long Qi

**Affiliations:** 1 Nursing School of Jilin University, Changchun, China; 2 Department of the Lymphatic and Vascular Surgery, China-Japan Union Hospital of Jilin University, Changchun, China; University Magna Graecia of Catanzaro, ITALY

## Abstract

The study aims to explore the comprehensive reasons for patients’ non-compliance with graded elastic compression stockings (GECS) as the treatment for lower limb varicose veins. Phenomenological analysis was applied in this qualitative study. The patients diagnosed with lower limb varicose veins and undergoing elective surgery who showed non-compliance with GECS as the treatment were invited to have semi-structured, in-depth, face-to-face interviews. Colaizzi method was employed to analyze the data for emerging themes associated with the reasons for patients’ non-compliance. Four main themes and nine subthemes related to the reasons for non-compliance with GECS for lower limb varicose veins were summarized. The main themes that emerged were (1) gaps in the knowledge of GECS therapy as a treatment for lower limb varicose veins, (2) few recommendations from the doctors and nurses, (3) disadvantages of GECS, and (4) sociopsychological factors. These themes provide data for policy and planning to improve patients’ compliance with GECS in China. Patients, healthcare professionals, and policy makers should share the responsibility to improve patients’ compliance with GECS therapy.

## Introduction

Lower limb varicose veins is a common condition and in China approximately 8.89% to 16.5% of the population is affected by it [1]. Graded elastic compression stockings (GECS) are the first-line treatment for lower limb varicose veins, since they provide the best overall results for patients [2]. Not only can GECS therapy delay disease progression, but it also improves the patients’ quality of life by maintaining the patients’ mobility. “Chinese experts’ consensus on the diagnosis and treatment of chronic venous diseases (CVD)” suggested that patients with CVD should undergo compression therapy throughout the treatment process [3].

Patients’ compliance is the cornerstone of GECS therapy. In fact, non-compliance with prescribed GECS is an apparent major cause of treatment failure. Unfortunately, patients affected by lower limb varicose veins tend to display low compliance with GECS therapy. Raju reported that 63% of patients did not comply with the prescribed regulations after using GECS for a period of time [4]. According to Ziaja’s study, which included a total of 16,770 patients diagnosed with CVD, 25.6% of whom used GECS as a treatment, 46.6% of the patients were never prescribed GECS therapy [5]. Another study reported that 29.2% of the patients were wearing GECS, but only 10.4% did so on a daily basis [6]. In addition, Inasi found that only 11.5% of the patients who were prescribed compression stockings used them regularly [7].

Poor compliance can exacerbate disease progression caused by varicose veins in the lower extremities, such as secondary edema and ulcers, among others. Kapp found that the participants who did not adhere were nine times more likely to have their wound to recur [8]. A randomized controlled trial found that non-compliant patients with healed venous leg ulcers were at a significantly greater risk of venous leg ulcers recurrence [9].

The medical effectiveness of GECS therapy as a treatment for lower limb varicose veins has been demonstrated using clinical trials. However, the success of GECS treatment largely depends on patients’ compliance. Unfortunately, data on patients’ compliance with GECS therapy are lacking in China. Previous research studies on the reasons why patients display poor compliance with GECS are mostly quantitative: questionnaires with limited options were used to choose and narrow the range of factors and thus yielded limited results. In Rastel’s study, only 10 factors were set for non-adherence to GECS therapy in non-complicated primary varicose veins when patients stopped wearing compression [6], while there were only four reasons for GECS non-compliance which were stated in another study [9]. The reasons for patients’ non-compliance with compression stockings as a treatment for lower limb varicose veins are multidimensional and complicated, and furthermore, investigations into comprehensive factors influencing compliance with GECS are limited, thus further investigations are needed [10].

To that end, the present study used a qualitative research method to comprehensively explore the factors affecting patients’ compliance with GECS for the treatment of lower limb varicose veins.

## Materials and methods

Phenomenological analysis was applied in this qualitative study whose aim is to understand the meaning and essence of participants’ experiences in a specific situation. We try to understand the subjective experiences of participants by returning to the situation itself. In the phenomenological approach, the situation itself refers to the subjective feelings, perceptions, and reactions experienced by the participants in a specific life scene [11]. The study followed a descriptive qualitative design using semi-structured, in-depth, face-to-face interviews to explore the reasons for non-compliance with GECS in patients diagnosed with lower limb varicose veins at the China-Japan Union Hospital of Jilin University from September to October, 2019. This method was chosen as it increased the understanding of the many reasons affecting patients’ compliance with GECS therapy and will help to develop qualitative tools and categories of inquiry in the future.

Purposive sampling was used to recruit participants who were representative of non-compliance with GECS [12]. The participants were patients who had been affected by lower limb varicose veins above ten years and were once recommended to use GECS to avoid aggravation of the condition. Additionally, they all had a history of low adherence to GECS treatment, which led to their condition worsening and thus having to undergo an elective surgery, as documented by their physicians. The inclusion criteria are listed in Table 1. The sample size was determined according to the saturation principle. Saturation principle should be measured from the perspective of interviewees and researchers. From the perspective of the interviewees, if the interviewees have finished their words or have no relevant content to express, the information has been saturated. From the researcher’s point of view, if the researcher is unable to gather new information and the information obtained is sufficient to analyze objective of his research, then the information is saturated [13]. The participants were to be representative in terms of gender, age, education, and type of medical insurance.

**Table 1:** Inclusion criteria.

No.	Inclusion criteria
1	The patients were 18 years old or older.
2	The patients had been affected by lower limb varicose veins above ten years.
3	The patients had been recommended to wear GECS previously.
4	The patients had the ability to wear GECS independently without any assistance.
5	The patients had no contraindications to GECS such as peripheral artery disease.
6	The patients had no cognitive dysfunction or language disorder.

Concerning compliance with GECS as a treatment for CVD, we developed a preliminary draft of our interview profile, because there were no such studies previously. Additionally, vascular surgery and nursing experts (a chief physician, an associate chief physician, a chief nurse, and a nurse-in-charge) were consulted. After pre-interviewing three participants, we revised the preliminary draft and created the final questions as shown in Table 2.

**Table 2:** The interview outline.

No.	Questions
1	How old were you when you found the first varicose veins in your lower limbs?
2	Did you wear GECS as prescribed?
3	Do you know which compression level of GECS you should wear?
4	Did you know the benefits of wearing GECS for your disease?
5	Do you know the right way to put on and take off GECS?
6	Why did you not wear GECS as prescribed?

The authors were all trained in conducting qualitative research and interviews prior to this study. Participants were assigned a code to ensure anonymity, and the interviews were conducted in a single, and quiet room. In general, the interviews took between 20 and 30 minutes and they were audiotaped. The interviewers were instructed to react neutrally to the participants’ answers and to try to remain sensitive and unbiased. Data collection stopped when recurrent patterns became evident in the participants’ narrations and data saturation was reached [12]. The audiotapes were transcribed into text within 48 hours of the interview, and later, the transcriptions were verified with the participants to ensure the authenticity of the data.

Data were collected in hospitals, and they were analyzed based on the phenomenological methodology suggested by Colaizzi [14]. The Colaizzi method was used to analyze the data and to find the themes and subthemes that emerged from the interviews. Colaizzi method has seven steps, including (1) familiarization, (2) identifying significant statements, (3) formulating meanings, (4) clustering themes, (5) developing an exhaustive description, (6) producing the fundamental structure, and (7) seeking verification of the fundamental structure [14,15].

This study was focused on clinical realities, and was consistent with the Consolidated Criteria for Reporting Qualitative Research checklist. All participants provided informed verbal consent using a standard script prior to the interviews. Furthermore, the research project was approved by the Ethics Committees of China-Japan Union Hospital of Jilin University (No.2019082111).

## Results

### Participant characteristics

Ten patients diagnosed with lower limb varicose veins and undergoing elective surgery were recruited. The mean age of the participants was 56.2 years (ranging from 34 to 69 years) and the length of time they had been affected by lower limbs varicose veins ranged from 3 to 36 years. Five (50.0%) were females who had each given birth. One patient (10.0%) had tertiary or higher education, and three (30.0%) had basic education. In addition, three of the participants (30.0%) had CVD in both legs, and four (40%) had CVD on the left leg only. Six (60%) came from urban settings and had social basic medical insurance, and only one participant, who was from a village, did not have any insurance. Full details of participants’ characteristics are described in Table 3.

**Table 3:** Participants’ characteristics.

Participant	Age	Gender	Education	Occupation	CEAP Grade	Site of VaricoseVeins	Insurance type
**A**	34	Female	Primary school	Worker	C4	Both	City
**B**	52	Female	Technical secondary school	Accountant	C3	Left	City
**C**	56	Male	Junior high school	Worker	C4	Right	City
**D**	67	Female	Primary school	Peasant	C4	Right	Rural cooperative
**E**	63	Male	Junior high school	Worker	C3	Left	Province
**F**	55	Female	Junior high school	Teacher	C2	Both	Province
**G**	69	Male	Primary school	Peasant	C4	Left	Rural Cooperative
**H**	59	Male	Illiterate	Peasant	C3	Right	Self-paying
**I**	64	Female	Senior high school	Salesperson	C3	Left	City
**J**	43	Male	College	Government employee	C2	Both	Province

### Emerging themes

The qualitative analysis revealed nine subthemes and four main themes, as shown in Table 4. The main themes include: (1) gaps in the knowledge of GECS therapy as a treatment for lower limbs varicose veins, (2) few recommendations from the doctors and nurses, (3) GECS’s disadvantages, and (4) sociopsychological factors. Each of these themes is explained below, including quotations from participants with their identification code.

**Table 4:** Main themes and subthemes identified.

Main Themes	Subthemes
Gaps in the knowledge of GECS therapy as a treatment for lower limb varicose veins.	Varicose veins is not a serious disease.
	What is GECS and how to use it?
	GECS is useless.
Few recommendations from the doctors and nurses.	Non-standardized prescription and management of GECS.
	Insufficient health education about lower limbs varicose veins and GECS therapy.
Disadvantages of GECS.	Putting on GECS is too difficult.
	Wearing GECS is too uncomfortable.
Sociopsychological factors.	GECS is so expensive.
	GECS is ugly.

#### Theme 1: Gaps in the knowledge of GECS therapy as a treatment for lower limb varicose veins

The participants selected for this study had both convergent and divergent perceptions regarding GECS therapy for varicose veins, which were reflected in the subthemes “Varicose veins is not a serious disease,” “What is GECS and how to use it?” and “GECS is useless.”

*Subtheme 1*: *Varicose veins is not a serious disease*. There was a convergent cognition that varicose veins was caused by prolonged standing, which was not seen as a threat to life among the participants, particularly among the subjects whose jobs involved standing for long periods of time as they stated. It was found that condescension for varicose disease reduces compliance with GECS.

*“I’m a teacher*, *and I think the lower limbs varicose veins may be caused by my job*. *I’ve heard that elastic stockings are a means of healthcare*, *but I don’t have any uncomfortable feelings.”*

(Patient F)

*“I have been suffering from the lower limbs varicose veins for more than 20 years*. *At first*, *only a few veins protruded from the surface of the leg*. *The doctor had told me to wear GECS* *previously*, *but my legs were not in pain or swollen at that time*, *and I did not comply with the doctor’s advice to wear GECS.”*

(Patient D)

*Subtheme 2*: *What is GECS and how to use it*? Most of the patients did not know the classification of pressure, type, size, and material of GECS. There was confusion about how to put them on and take them off, the length of time they should be worn, the cleaning method, and the replacement time of GECS.

*“The doctor asked me to wear elastic socks*, *so I went to the pharmacy and bought a pair*. *But I don’t know how many types of socks there are and the differences between them*. *I’m not sure I bought the right ones.”*

(Patient B)

*Subtheme 3*: *GECS is useless*. GECS therapy is considered to be the first line treatment for CVD, even though it is a long-term process that requires patients to wear them all their lives. However, some participants thought that GECS had no effect on CVD. Others thought the pressure was not sufficient and the symptoms were not relieved quickly enough. Therefore, the perceived therapeutic effect of GECS affects patients’ compliance.

*“Are these elastic stockings really useful for the varicose veins*? *I used to wear a pair of elastic stockings for a while which felt really bad*. *The effect was not obvious*, *therefore I stopped wearing it.”*

(Patient A)

#### Theme 2: Few recommendations from the doctors and nurses

*Subtheme 4*: *Non-standardized prescription and management of GECS*. Most of the interviewed participants said that their doctors did not describe GECS in detail, and the doctors or nurses usually told them about it verbally. Only a few healthcare practitioners were actually encouraging patients to try GECS.

*“The doctor always told me to wear elastic stockings*, *but he didn’t give me any prescription*, *nor did the doctors and nurses patiently told me how to choose or wear the elastic stockings*. *The elastic stockings that I bought were always at the bottom of the drawer.”*

(Patient J)

*Subtheme 5*: *Insufficient health education about lower limb varicose veins and GECS therapy*. Based on our findings, we believe that it should be mandatory for doctors and nurses to educate patients about CVD and GECS. This would help patients understand the treatment better. However, due to heavy clinical work, the doctors and nurses usually had little time for health education.

*“The doctors and nurses would be so busy all day*, *I generally asked my children to search for information online*, *and tried my best not to bring too much trouble to doctors and nurses.”*

(Patient C)

#### Theme 3: Disadvantages of GECS

*Subtheme 6*: *Putting on GECS is too difficult*. In order to guarantee the effectiveness of compression therapy, GECS is made of special materials in a unique production process that adds higher pressure and length to the thigh which bring difficulties to the patients. With the increased pressure level of GECS, the difficulties would also increase.

*“I am old and my hands and feet are not flexible*. *It takes a lot of effort to put on the stockings*, *so I would not wear them.”*

(Patient J)

*Subtheme 7*: *Wearing GECS is too uncomfortable*. When wearing the GECS, patients might suffer from compression side effects, such as burning, sweating, itching, pain, and allergies amongst others. This would make patients uncomfortable and lead to them giving up the GECS.

“In winter, I can usually wear GECS as required, but in summer they are too hot to wear them.”

(Patient G)

*“It’s hard to wear stockings on the ankle*, *so I cut off the portion of the foot myself*, *and the rest will be enough to hold the calf.”*

(Patient I)

#### Theme 4: Sociopsychological factors

*Subtheme 8*: *GECS is so expensive*. Due to the special materials used to make graduated compression stockings, the price of GECS is usually high, especially when they are imported ones, and further, urban and rural basic medical insurance does not cover GECS therapy. Therefore, patients with low income, such as farmers, would be unable to afford the expensive treatment. Due to being under great psychological pressure to reduce the financial burden of their families, patients would usually give up on wearing GECS.

*“A pair of stockings sells for several hundred yuan*. *It’s too expensive without medical insurance.”*

(Patient H)

*Subtheme 9*: *GECS is ugly*. Some interviewees thought that GECS had an unaesthetic appearance, therefore, they were not willing to wear them. In addition, some male patients who had traditional viewpoints believed that GECS were a women-specific attire and refused to wear them. Some patients were afraid that GECS would attract others’ attention, which represented a psychological pressure for them.

*“Sometimes when I was walking in the park*, *people would come up to me and ask me*: *what are your stockings for*? *Is there something wrong with you*? *They were getting on my nerves.”*

(Patient E)

## Discussion

This qualitative study explored the comprehensive factors influencing patients’ non-compliance with GECS as a treatment for lower limb varicose veins. The resulting factors are grouped into categories that include: (1) gaps in the knowledge of GECS therapy as a treatment for lower limb varicose veins, (2) few recommendations from doctors and nurses, (3) disadvantages of GECS, and (4) sociopsychological factors. These findings are of fundamental importance to clinicians and nurses who treat patients with varicose veins.

Results indicated that most patients lacked knowledge about lower limb varicose veins and GECS therapy, which led to poor compliance. Participants believe that varicose veins are not a serious disease, and they did not realize the danger associated with the condition worsening.

Some patients did not wear GECS, because they felt that their symptoms were not serious and that their varicose veins had no impact on their work, housework, or quality of life. Additionally, the majority of participants had no idea of the important role of GECS as a treatment for lower limb varicose veins. Further, since GECS is a long-term treatment and thus did not show immediately improvements, patients complained that GECS did not relieve their symptoms quickly enough.

As the results revealed, there is poor awareness of lower limb varicose veins and GECS therapy in the Chinese general population. This follows from the existence of insufficient educational programs about GECS therapy in hospitals. This was consistent with Apenteng’s study that reported that patients were less educated about varicose veins, and that health education about GECS was often perfunctory [16]. Kristina Heyer also reported that there was much lower knowledge and practical skills about compression therapy than expected [17].

One of the most relevant factors for compliance is patients’ health education and motivation [18]. Uhl suggested that practitioners’ recommendations are important for better compliance [19]. Learning from example, the Japanese Society of Phlebology established a qualification system for elastic stocking conductors to promote proper usage of GECS in 2002. GECS experts are currently working in outpatient clinics to increase compliance with GECS by meticulous consultation with patients regarding compression therapy [20]. We should learn from Japan’s experience and establish educational programs to increase patients’ compliance with GECS as soon as possible.

This study also revealed that there is a lack of awareness about GECS therapy among the clinicians and nurses, which resulted in limited educational programs. In fact, a study reported that knowledge about GECS among Chinese healthcare professionals was poor [21]. Although “Chinese experts agree on the diagnosis and treatment of chronic venous diseases”, suggesting that patients with CVD should accept compression therapy throughout the whole process [3], there are no specific guidelines about GECS therapy.

Moreover, there is not a clear division of responsibilities in the medical system regarding GECS therapy. While it should be mandatory for doctors and nurses to provide education for patients, they have little time for health education due to their heavy clinical work. Thus, medical institutions should take this situation seriously and provide training for clinically applying GECS among healthcare professionals [19].

In addition, we should reinforce to develop more complete guidelines of health education about the use of GECS based on both medical and practical points of view [5]. A series of standardized procedures for the treatment of lower limbs varicose veins with GECS therapy should be contained in the guidelines, including doctor’s prescription, measurement, fitting, follow-up, and so on.

Beyond that, educational programs led by nurses responsible for sizing and applying GECS and teaching patients the correct usage of GECS are necessary [22]. Professional and standardized management of GECS can help increase the patients’ trust in the treatment. The more the patients believe in the effectiveness of the treatment, the more they will comply with GECS use.

Furthermore, healthcare practitioners should prescribe GECS and take into consideration patients’ individual differences, including diagnosis, pressure level, quantity, and length. Above all, more attention should be paid to compliance, which is vital for a treatment’s success, and in particular, the differences between pressure levels and the length of GECS should be studied [21]. Patients’ compliance with lower level of GECS is likely to be higher than compliance to high levels, which suggests that clinicians should consider prescribing lower levels GECS and assess compliance before increasing compression levels.

The GECS have several disadvantages as well. To ensure the effectiveness of compression therapy, GECS are designed to have higher pressure and length [23]. When the level of pressure of GECS increases, the difficulties in wearing them will increase, and result in non-compliance. In fact, a study from Brazil showed that the main reason for non-compliance with GECS was difficulties when putting them on [24]. This could be simply remedied by prescribing donning aids which could increase success rates significantly. Sippel found that the success rate of using donning devices to put on one 40 mmHg GECS was 88%. However, without donning devices, success was 60%. Not only can donning aids improve the treatment’s success rate and patients’ compliance with GECS, but they can also increase the patients’ independence, self-efficacy, and quality of life [25]. Chinese nurses could select appropriate donning aids taking into account the patients’ individual differences and teach them how to use these aids.

On the other hand, patients might suffer from GECS side effects such as burning, sweating, itching, pain, and allergies amongst others, which would reduce patients’ comfort and compliance with GECS. Rastel found that 32.6% of patients did not wear their compression stockings mainly because they could not tolerate them well [6]. A summation analysis of complications of GECS raised that skin irritation was a common event [10]. A study on the quality of patients’ view of compression therapy also raised the main side effects were dryness of skin, itching, slipping, or constriction [26]. This finding should persuade doctors to recommend accurate prescriptions of GECS by proper measuring, fitting of GECS and addressing the personal and specific needs of the patient to enhanced concordance. Manufacturers also should share the responsibility to look for alternative materials for production to maximize comfort and aesthetic acceptance [27].

Regarding socioeconomic factors, it was found that exorbitant prices bring economic pressure to the patients: the high cost of GECS is a common reason for discontinuation of compression therapy [5]. In Britain, Germany, and other European countries, GECS are listed as one of the statutory tools covered by medical insurance. Meanwhile, Chinese medical insurance is not responsible for covering GECS therapy. Therefore, it is suggested to include GECS therapy in the scope of basic medical insurance reimbursement.

The effectiveness of GECS treatment largely depends on the patients’ compliance. However, compliance is hard to assess, as the literature shows. Allaert developed a validated, short, self-administered questionnaire to evaluate the patients’ adherence to the GECS treatment. Physicians and nurses should use this suitable tool to assess patients’ compliance with GECS in their day-to-day practice [28]. In the future, the methods for measuring compliance should also be unified, and medical staff should pay attention to the evaluation of patients’ compliance. At the same time, nurses should develop new methods to evaluate and improve compliance, especially outside the hospital setting [29,30].

There is limited research about the comprehensive factors influencing compliance with GECS therapy for lower limb varicose veins. This is the first qualitative study that explored patients’ attitudes and reasons for non-compliance with GECS therapy from the patients’ perspective by conducting in-depth, face-to-face interviews in the northeast of China to propose effective and targeted management strategies. There are limitations to our purposive sampling that may bias our findings, such as overrepresentation of surgical patients and those with lower educational levels and low compliance.

## Conclusions

Poor adherence to treatment is multi-factorial and it was broadly grouped into categories in this study. Based on our findings, we believe it is necessary to establish guidelines about GECS therapy and the associated education programs in China as soon as possible. The medical insurance system should be updated to include coverage of GECS therapy as well. Doctors and nurses should contribute by raising awareness and providing education regarding GECS therapy. Furthermore, the compression stockings’ manufacturers could look for other materials that might be more comfortable for patients. In general, patients, healthcare professionals, and policy makers should share the responsibility to improve patients’ compliance with GECS therapy.

## Supporting information

S1 File (DOCX)

S1 Table (RAR)
