# Supplementary material for: Reasons for patient non-compliance with compression stockings as a treatment for varicose veins in the lower limbs: A qualitative study
Source: PLoS One. 2020 Apr 28;15(4):e0231218. doi: 10.1371/journal.pone.0231218 (PMC7188228; doi:10.1371/journal.pone.0231218)
Supplement: S1 File — (DOCX) [file pone.0231218.s001.docx]

**Supporting information: Partial interview data of the participants**

**Partial interview data of Patient A**

Q1: How old were you when you found the first varicose veins in your lower limbs?

Patient A: 18 or 19 years old.

Q2: Did you wear GECS as prescribed?

Patient A: No. I used to wear a pair of elastic stockings for a while. However I prefer the skinny stockings that girls wear.

Q3: Do you know which compression level of GECS you should wear?

Patient A: I have no idea.

Q4: Did you know the benefits of wearing GECS for your disease?

Patient A: Doctors said that it can slow the progress of the disease.

Q5: Do you know the right way to put on and take off GECS?

Patient A: Is there anything special? Just wear them like normal stockings.

Q6: Why did you not wear GECS as prescribed?

Patient A: Are these elastic stockings really useful for the varicose veins? I used to wear a pair of elastic stockings for a while which felt really bad. The veins protruding from my leg did disappear when I put the GECS on, but when I took them off, the veins were still protruding. The effect was not obvious, therefore I stopped wearing it.

Further more, the silicone yarn on the top band of elastic stockings that is designed to keep it in place will make my skin red and itchy. Maybe I am allergic to that stuff.

The compression stockings are ugly and look like a proprietary thing of the elderly. When I am wearing them, I feel I am an old person. I prefer the skinny stockings that girls wear.

**Partial interview data of Patient B**

Q1: How old were you when you found the first varicose veins in your lower limbs?

Patient B: I've had varicose veins since I was younger, I'm not sure exactly age, approximately in my twenties.

Q2: Did you wear GECS as prescribed?

Patient B: No.

Q3: Do you know which compression level of GECS you should wear?

Patient B: I do not know.

Q4: Did you know the benefits of wearing GECS for your disease?

Patient B: I used to work as an accountant, and I usually sat down a lot almost every day. It is said that elastic stockings are good for the people who often sat, which could prevent varicose veins. I also learned that elastic stockings were good for varicose veins from the doctor. Maybe it can cure this varicose vein.

Q5: Do you know the right way to put on and take off GECS?

Patient B: It is the same with regular stockings.

Q6: Why did you not wear GECS as prescribed?

Patient B: Last year, I went to the hospital for the varicose veins and the doctor told me to have an operation, but I didn't want to have an operation at that time. Then the doctor asked me to wear elastic stockings, so I went to the pharmacy and planned to bought a pair. But when I got there, I found there were so many types and brands and I had no idea of the differences between them. I was not sure I bought the right stockings since I purchased the ones that were recommended by the pharmacy’s salesclerk.

The doctors and nurses did not tell me what kind of elastic stockings to buy, only that they were medical elastic stockings. I also want to ask the doctors and nurses for more information, but they were too busy and I didn't bother them. In fact, I also had a lot of questions about the elastic stockings. For instance, whether only the limb with varicose veins needed the elastic stockings, or both limbs.

When wearing the elastic stockings, I felt very uncomfortable because that the elastic stockings tightly around my legs. I just wore them for a few days. As a result, this varicose vein became serious again this year. I felt my leg was very heavy and a little painful. When I came to the hospital again, the doctor said I must have an operation, so I did.

**Partial interview data of Patient C**

Q1: How old were you when you found the first varicose veins in your lower limbs?

Patient C: I had a family history of varicose veins in the home, my mother and my aunts have the varicose veins, when I was a young man I also found that the varicose veins.

Q2: Did you wear GECS as prescribed?

Patient C: I only wear it for a few days.

Q3: Do you know which compression level of GECS you should wear?

Patient C: I do not know.

Q4: Did you know the benefits of wearing GECS for your disease?

Patient C: Can elastic stockings cure varicose veins?

Q5: Do you know the right way to put on and take off GECS?

Patient C: I learned from a video of wearing elastic stockings which my son found from the Internet. I'm not sure.

Q6: Why did you not wear GECS as prescribed?

Patient C: When I was young I also found that the varicose veins, but the time is not too serious, so I didn't treat, I went to the hospital to see a doctor, the doctor said it was not serious, and let me wear elastic stockings, but he did not tell me buy which kind. There were a lot of people, the doctor was very busy, so I did not continue to ask the doctor. I generally asked my children to search for information online, and tried my best not to bring too much trouble to doctors and nurses.

My son bought me a pair of elastic stockings from internet, and I wore them for a few days because of so many difficulties to put on them. It took me a lot of time to put on and take off my stockings every day which cost me too much effort. In addition, when I put on stockings, I feel very uncomfortable. I always feel my legs are too tight and itchy.

Further, the elastic stockings are similar to the silk stockings worn by women. Every time I put on the elastic stockings, I must wear trousers. I am afraid of being seen by others. Therefore I stopped wearing stockings.

**Partial interview data of Patient D**

Q1: How old were you when you found the first varicose veins in your lower limbs?

Patient D: About 40 years old.

Q2: Did you wear GECS as prescribed?

Patient D: No.

Q3: Do you know which compression level of GECS you should wear?

Patient D: I do not know.

Q4: Did you know the benefits of wearing GECS for your disease?

Patient D: The doctor said that the elastic stockings are good for my disease, I don't know exactly what benefits.

Q5: Do you know the right way to put on and take off GECS?

Patient D: I do not know.

Q6: Why did you not wear GECS as prescribed?

Patient D: I have been suffering from the lower limbs varicose veins for more than 20 years. At first, only a few veins protruded from the surface of the leg. The doctor had told me to wear GECS [previously](javascript:;), but my legs were not in pain or swollen at that time, and I did not comply with the doctor’s advice to wear GECS.

In our countryside, we often work in the fields and do some heavy manual labor. A number of people have the lower leg varicose veins, but there is nobody wearing elastic stockings. I also heard that the price of elastic stockings is quite high. A pair of elastic stockings costs me enough money for a month food, and I am reluctant to buy them.

Now my leg symptom of vein varicose is serious, such as the skin has blackened. I do not know whether because I did not listen to the doctor's advice. If I had been wearing the elastic stockings, it might not have been so bad. I wouldn't have needed surgery.

**Partial interview data of Patient E**

Q1: How old were you when you found the first varicose veins in your lower limbs?

Patient E: I had varicose veins in my twenties.

Q2: Did you wear GECS as prescribed?

Patient E: I used to wear it for a while, but then I stopped.

Q3: Do you know which compression level of GECS you should wear?

Patient E: I remember the elastic stockings I wore at that time were grade tow.

Q4: Did you know the benefits of wearing GECS for your disease?

Patient E: Wearing the elastic stockings, I felt indeed a little more comfortable than not wearing. I felt the legs were not so heavy anymore. There were no prominent veins.

Q5: Do you know the right way to put on and take off GECS?

Patient E: The doctor has ever told me how to wear elastic stockings. First the leg part of the elastic stockings are took off down, and then the foot part of the elastic stockings are put on, at last the leg part of the elastic stockings are pulled up.

Q6: Why did you not wear GECS as prescribed?

Patient E: The doctor told me to buy elastic stockings myself. The first time I bought a pair of stockings which were too tight, so I bought another pair of large ones for comfort. I had varicose veins in only one leg, but the doctor told me to wear stockings on both legs. I also did not follow the doctor's advice and usually wore one stockings on one leg.

In addition, concerns regarding elastic stockings appearance was troubled me. I would not wear elastic stockings with shorts or dresses. Sometimes when I was walking in the park, people would come up to me and ask me: what are your stockings for? Is there something wrong with you? They were getting on my nerves.

Another reason is that with age my hands and feet are not flexible. It takes a lot of effort to put on the stockings, so I would not wear them.

I like sports very much. I walk in the park every day. I can walk 5 kilometers every day. Of course, I have not worn stockings when I exercise these days. Recently, when walking I felt very painful in my leg. I predicted that the varicose veins were aggravated. When I went to the hospital for examination, the doctor said that the varicose veins were serious and suggested me having a surgery as a treatment.

**Partial interview data of Patient F**

Q1: How old were you when you found the first varicose veins in your lower limbs?

Patient F: About 35 years old.

Q2: Did you wear GECS as prescribed?

Patient F: No.

Q3: Do you know which compression level of GECS you should wear?

Patient F: I do not know.

Q4: Did you know the benefits of wearing GECS for your disease?

Patient F: Elastic stockings seem to promote blood circulation, but I am not sure exactly. The main expected effects of elastic stockings are the decrease of symptoms and the prevention of progression of the disease.

Q5: Do you know the right way to put on and take off GECS?

Patient F: Just like women wear stockings.

Q6: Why did you not wear GECS as prescribed?

Patient F: I am a teacher, and I think the lower limbs varicose veins may be caused by my job. Because I have to stand at work, sometimes for more than eight hours a day. Blood was gathered because of standing for long periods of time in the lower limbs, so varicose veins developed. From the outside you can see veins protruding, like an earthworm, but I do not have any uncomfortable feelings. Because this varicose vein makes the leg look really ugly, I usually wear trousers to avoid exposing my legs. I have heard that elastic stockings are a means of healthcare. The varicose veins in the lower limbs disappeared when I was wearing the elastic stockings. However, if I took the stockings off, the veins still bulged out. The elastic stockings only temporarily alleviate the symptoms, and have no therapeutic effect on varicose veins. Some people say that after wearing elastic stockings the legs are very comfortable, but I feel very tight on my leg with the elastic stockings, very uncomfortable, and very hot in summer. I wore my elastic stockings for one year, and then the stockings did not look like as elastic as they used to. I felt useless, and I did not buy new elastic stockings. I did not wear them anymore since then.

Last year, I felt the symptoms of varicose veins in my legs became worse. It was not painful before, but recently I felt pain, and then there was a special bulge. I came to the hospital to see a doctor this time, the doctor said that I have already appeared the complication of varicose veins, and they must give the operation to me.

**Partial interview data of Patient G**

Q1: How old were you when you found the first varicose veins in your lower limbs?

Patient G: About sixties.

Q2: Did you wear GECS as prescribed?

Patient G: No. My daughter bought me a pair of elastic stockings to wear, but I can not stand them.

Q3: Do you know which compression level of GECS you should wear?

Patient G: I don't know.

Q4: Did you know the benefits of wearing GECS for your disease?

Patient G: Everyone says that wearing elastic stockings has profit, but I do not know specific benefit.

Q5: Do you know the right way to put on and take off GECS?

Patient G: I do not know.

Q6: Why did you not wear GECS as prescribed?

Patient G: Varicose veins are said to be caused by prolonged standing or sitting. I used to work in the field a lot, but I did not see varicose veins then. However now I do not work, and I have been suffering the varicose veins. The doctor advised me to wear the elastic stockings, but the elastic stockings were too difficult to put on and take off. If you wear elastic stockings, you will also feel very uncomfortable and trouble. It is because it is very difficult to put on and take off elastic stockings every day I do not like wearing them. In addition, the stockings are too tight to fit easily. In winter, I can usually wear GECS as required reluctantly, but in summer they are too hot to wear them. I have wore elastic stockings for half a year, and I could not insist on continuing to wear elastic stockings.

**Partial interview data of Patient H**

Q1: How old were you when you found the first varicose veins in your lower limbs?

Patient H: About ten years ago.

Q2: Did you wear GECS as prescribed?

Patient H: No.

Q3: Do you know which compression level of GECS you should wear?

Patient H: I do not know.

Q4: Did you know the benefits of wearing GECS for your disease?

Patient H: The elastic stockings have advantage on vein varicose. It is said that the leg would be not swollen and varicose vein is disappeared.

Q5: Do you know the right way to put on and take off GECS?

Patient H: Is not it the same as wearing socks?

Q6: Why did you not wear GECS as prescribed?

Patient H: I am a farmer, and I earn very little a year by farming. A pair of stockings sells for several hundred yuan. The doctor suggested to buy two pairs of stockings for change. It is too expensive without medical insurance. Besides, varicose veins are not going to kill me, so I am just going to hang on.

The varicose veins will not threaten my life, and I have no pain and swelling feeling. However the doctor said my varicose veins have been very serious, and there were related complications, such as there was skin hardening. My leg must be operated as soon as possible.

**Partial interview data of Patient I**

Q1: How old were you when you found the first varicose veins in your lower limbs?

Patient I: More than 50 years old.

Q2: Did you wear GECS as prescribed?

Patient I: Sometimes I wear elastic stockings, sometimes I don't.

Q3: Do you know which compression level of GECS you should wear?

Patient I: I do not know which pressure level the stockings I wore were. Maybe they are class I. I put on the compression stockings as common thigh stockings, but they were more difficult than common stockings.

Q4: Did you know the benefits of wearing GECS for your disease?

Patient I: When you put on the elastic stockings, the blood of the lower legs could flow back to the heart faster, and the symptoms of varicose veins will be much less.

Q5: Do you know the right way to put on and take off GECS?

Patient I: I see. The doctor has ever told me. I have also seen how to wear elastic stockings on TV shows.

Q6: Why did you not wear GECS as prescribed?

Patient I: It is hard to wear stockings on the ankle, because there is too much pressure on the ankle. I am a little fat and there is difficult to bend over to wear the elastic stockings.

I cut off the portion of the foot myself, and the rest will be enough to hold the calf. It is easier to wear this way.

**Partial interview data of Patient J**

Q1: How old were you when you found the first varicose veins in your lower limbs?

Patient J: About 30 years old.

Q2: Did you wear GECS as prescribed?

Patient J: I used to wear elastic stockings, but I didn't stick with wearing them.

Q3: Do you know which compression level of GECS you should wear?

Patient J: I have look it up on the Internet myself, and level 2 pressure may be suited for me in this situation.

Q4: Did you know the benefits of wearing GECS for your disease?

Patient J: The pressure of elastic stockings gradually decreases from the ankle up to the knee. Elastic stockings speed up the return of venous blood, so that blood does not accumulate in the legs and the veins don't stick out.

Q5: Do you know the right way to put on and take off GECS?

Patient J: I also look up videos on the way to put on and take off GECS from the Internet. I need to put a slider in the front of the foot, turn the leg part of elastic stockings over and put the foot part on at first and then put the leg part on. Finally, remove the slide.

Q6: Why did you not wear GECS as prescribed?

Patient J: The doctor always told me to wear elastic stockings, but he didn't give me any prescription, nor did the doctors and nurses patiently told me how to choose or wear the elastic stockings. The elastic stockings that I bought were always at the bottom of the drawer.

Elastic stockings are really uncomfortable when they are put on the legs. They are very tight. In winter, the skin could be itched and dried by the elastic stockings. In summer, the weather is so hot, wearing the elastic stockings on the legs will be more hot. Anyway, it is just too hard to wear elastic stockings. I can't insist on wearing stretch stockings all the time.
