# Supplementary material for: Reasons for patient non-compliance with compression stockings as a treatment for varicose veins in the lower limbs: A qualitative study
Source: PLoS One. 2020 Apr 28;15(4):e0231218. doi: 10.1371/journal.pone.0231218 (PMC7188228; doi:10.1371/journal.pone.0231218)
Supplement: Attachment — Submitted filename: Response to Reviewers.docx [file pone.0231218.s003.docx]

**PONE-D-19-34825，entitled “Reasons for patients’ non-compliance with compression stockings as a treatment for varicose veins in the lower limbs’: A qualitative study”**

Dear Prof. Raffaele Serra,

Thank you for your letter and for the reviewers' comments concerning our manuscript. We apologize for my late return of our revised manuscript, because our country suffered from severe epidemic—— novel coronavirus pneumonia which brought us a lot of difficulties, so we hope you could accept our apology and give us another chance.

According to the comments and suggestions, we have revised the relevant parts of the manuscript and are resubmitting the manuscript. We also responded point by point to the comments from the editor and the reviewers as shown below, and all changes are marked in red in the revised version of the manuscript.

Thank you very much for considering our revised manuscript. We look forward to hearing from you at your earliest convenience.

With kindest regards,

JianMei Gong, JianShi Du, DongMei Han, Xinyu Wang, and Shaolong Qi

Responses to Journal Requirements:

When submitting your revision, we need you to address these additional requirements:

1. Please ensure that your manuscript meets PLOS ONE's style requirements, including those for file naming. The PLOS ONE style templates can be found at http://www.plosone.org/attachments/PLOSOne_formatting_sample_main_body.pdf and http://www.plosone.org/attachments/PLOSOne_formatting_sample_title_authors_affiliations.pdf

Response: We guarantee that manuscript meets PLOS ONE's style requirements, including those for file naming.

2. Please amend your current ethics statement to address the following concerns: Please explain why was written consent was not obtained, how you recorded/documented participant consent, and if the ethics committees/IRBs approved this consent procedure.

Response: The ethics statement of this study has been described in 109-112. All participants provided informed verbal consent using a standard script prior to the interviews. Furthermore, the research project was approved by the Ethics Committees of China-Japan Union Hospital of Jilin University (No.2019082111).

3. Please amend the manuscript submission data (via Edit Submission) to include author DongMei Han, Xinyu Wang and Shaolong Qi

Response: We have amended the manuscript submission data (via Edit Submission) to include author DongMei Han, Xinyu Wang and Shaolong Qi.

4. Please include captions for your Supporting Information files at the end of your manuscript, and update any in-text citations to match accordingly. Please see our Supporting Information guidelines for more information: <http://journals.plos.org/plosone/s/supporting-information>.

Response: We have included captions for the Supporting Information files at the end of the manuscript, and update any in-text citations to match accordingly. In addition, we have made some changes to the Table 1, Table 2 and Table 3 as shown in red.

Responses to the reviewer # 1

[Note: HTML markup is below. Please do not edit.]

Reviewers' comments:

Reviewer's Responses to Questions

Comments to the Author

1. Is the manuscript technically sound, and do the data support the conclusions? The manuscript must describe a technically sound piece of scientific research with data that supports the conclusions. Experiments must have been conducted rigorously, with appropriate controls, replication, and sample sizes. The conclusions must be drawn appropriately based on the data presented.

Reviewer #1: Partly

Response: Questionnaires with limited options which were used to fill by objects in previous studies so that there was narrow the range of factors. It is a basis of quantitative studies that a qualitative research method could comprehensively explore the factors affecting patients’ compliance with GECS for the treatment of lower limb varicose veins. So it is reasonable for us to choose this qualitative research method. Our study was focused on clinical realities which was consistent with the Consolidated Criteria for Reporting Qualitative Research checklist and the data support the conclusions powerfully.

________________________________________

2. Has the statistical analysis been performed appropriately and rigorously?

Reviewer #1: No

Response: Qualitative research is different from quantitative research where statistical analysis is not appropriate. Phenomenological analysis was applied in this qualitative study whose aim is to understand the meaning and essence of participants' experiences in a specific situation. The patients who had a non-compliance of GECS were interviewed in depth about their experiences regarding factors why they did not wear GECS. We try to understand the subjective experiences of participants by returning to the situation itself by phenomenology. Data were collected in hospitals, and it is appropriate and rigorous that Colaizzi method of phenomenological analysis was applied to analyze the data.

________________________________________

3. Have the authors made all data underlying the findings in their manuscript fully available?

The PLOS Data policy requires authors to make all data underlying the findings described in their manuscript fully available without restriction, with rare exception (please refer to the Data Availability Statement in the manuscript PDF file). The data should be provided as part of the manuscript or its supporting information, or deposited to a public repository. For example, in addition to summary statistics, the data points behind means, medians and variance measures should be available. If there are restrictions on publicly sharing data—e.g. participant privacy or use of data from a third party—those must be specified.

Reviewer #1: Yes

Response: Thank you for your encouragement.

________________________________________

4. Is the manuscript presented in an intelligible fashion and written in standard English?

PLOS ONE does not copyedit accepted manuscripts, so the language in submitted articles must be clear, correct, and unambiguous. Any typographical or grammatical errors should be corrected at revision, so please note any specific errors here.

Reviewer #1: Yes

Response: Thank you for your encouragement.

________________________________________

5. Review Comments to the Author

Please use the space provided to explain your answers to the questions above. You may also include additional comments for the author, including concerns about dual publication, research ethics, or publication ethics. (Please upload your review as an attachment if it exceeds 20,000 characters)

Response: I don't think we need a reply here.

Reviewer #1:

A. Overall evaluation and general comments

1. As I am quite skilled in quantitative experiments and almost a beginner in qualitative research, I am not the right person to judge the level of your qualitative research.

Anyway, the number of patients is very small (10) and paradoxically less than the sum of the number of themes(4) and subthemes(9). The more the number of categories, the more (much more) are the number of sets of categories, generally in a combinatorial way. This sets the issue of an adequate experimental design. For instance, even if considering just sex(M/F) and CEAP(1-6) the classes are 12, more than the available patients.

Response: It is unnecessary that the sample size is calculated in advance of the qualitative study. When the interviews were conducted till theoretical saturation was achieved such that no new contents appeared in the interviews and the same type of concepts and themes emerged in the data analysis, the sample is saturated.

2.The paper is missing any report about the qualitative-quantitative tools used to analyze gathered data. Thus, we leave quantitative research but we do not get any qualitative evaluation to help us in our judgements.

Response: Thanks for the suggestion. We might have omitted the description of this part in the materials and methods, so we have supplemented some report about the qualitative-quantitative tools in the manuscript. For example,

Phenomenological analysis was applied in this qualitative study whose aim is to understand the meaning and essence of participants' experiences in a specific situation. We try to understand the subjective experiences of participants by returning to the situation itself. In the phenomenological approach, the situation itself refers to the subjective feelings, perceptions, and reactions experienced by the participants in a specific life scene [11].

Data were collected in hospitals, and they were analyzed based on the phenomenological methodology suggested by Colaizzi [14].

3.The article is prolix in some sections. Why not to shorten the text, avoiding repetitions ?

Response: Thanks for the suggestion. We have erased the repetition in the text according to your recommendations as shown in the Revised Manuscript with Track Changes.

4.In order to shorten the text into a non-verbose and more clear one, provide just one example of patient interview instead of two.

Response: Thanks for the suggestion. We have deleted redundant example of patient interview for keeping one according to your recommendations as shown in the Revised Manuscript with Track Changes.

B. Detailed evaluation of specific deficiencies with suggestions for improvements

1.67 > We were able to identify four main reasons for patients noncompliance.,

Omit the sentence, as it goes in other sections or it is a repetition.

Response: Thanks for the suggestion. We have omitted the sentence according your recommendation.

2.75 > Purposive sampling, An intentional (purposive) sampling is just a selection by previous knowledge. Most people are not used to purposive sampling (opposed to probability sampling). Add therefore a reference.

Response: The purpose of sampling is usually applied in the qualitative research instead of random sampling. According to the purpose of the study, “To explore the comprehensive reasons for patients’ non-compliance with graded elastic compression stockings (GECS) as the treatment for lower limb varicose veins” we could only look for those patients with non-adherence to GECS, rather than those who have good adherence. We have added a reference according your recommendation.

12 van Hoeven LR, Janssen MP, Roes KCB, Koffijberg H. Aiming for a representative sample: Simulating random versus purposive strategies for hospital selection. BMC Med Res Methodol, 2015, 15:90 http://doi.org/10.1186/s12874-015-0089-8 PMID: 26497748

3.81 > saturation principle,

Response: Thanks for the suggestion. We have added a reference according your recommendation.

12 Pliuskevicius R. On the Saturation Principle for a Linear Temporal Logic. Computational Logic and Proof Theory, 1993, 713: 289-300. http://doi.org/10.1007/BFb0022577

4.91-95 Report the final questions in a table and delete them from the text

Response: Thanks for the suggestion. We accepted the recommendation and revised the manuscript as shown in the Revised Manuscript with Track Changes.

**Table 2. The interview outline.**

(1) How old were you when you found the first varicose veins in your lower limbs?

(2) Did you wear GECS as prescribed?

(3) Do you know which compression level of GECS you should wear?

(4) Did you know the benefits of wearing GECS for your disease?

(5) Do you know the right way to put on and take off GECS?

(6) Why did you not wear GECS as prescribed?

5.344-345 > GECS are listed as one of the statutory diseases covered by medical insurance, GECS are not a disease but a therapeutic tool, the corresponding disease is chronic venous disease (CVD)

Response: Thanks for the suggestion. I'm so sorry that we did not pay enough attention to that. We accept the recommendation and have changed “diseases” into “tools” as shown in the Revised Manuscript with Track Changes.

6.349 > However, compliance is hard to assess, as the literature shows.,

Recently wireless accelerometers were adopted to measure the wearing time of GECS. Look at the open access paper "Evaluation of an accelerometer-based device to monitor compliance on patients wearing medical compression stockings. E Grenier, D Rastel, C Chaigneau. JTAVR 2018;3(1):25-32. https://doi.org/10.24019/jtavr.51"

Response: Thanks for the recommendation. The study reported that integration of the system in an elastic compression stocking enables acquisition, analysis and interpretation of an acceleration signal thus providing objective monitoring of patient compliance as a function of the periods of wear and non-wear. But that study is still in the preliminary stage meanwhile there were 5 cases, so large-scale clinical studies were very limited. At present compliance is still hard to assess and we are also exploring this area.

C. Minor points e.g. grammar or terminology

1.8, 102, 104 > Colaizzi's method and Colaizzi method,

Decide how to refer to the method and use always the same way.

Response: We have decided use “Colaizzi method” after confirmation which was revised uniformly in the manuscript.

2.29 > condition,, and, in China, approximately,

condition and in China approximately

Response: Thanks for the suggestion. We accepted the recommendation and revised the manuscript as shown in red.

3.42-43 > Another study reported that although 29.2% of the patients were wearing GECS, but only 10.4% did so on a daily basis [6].,

You have 2 choices: omit "although" or omit "but". They cannot be present together.

Response: Thanks for the suggestion. We accepted the recommendation and revised the manuscript as shown in red.

4.48 > recur,

to recur

Response: Thanks for the suggestion. We accepted the recommendation and revised the manuscript as shown in red.

5.49 > found non-compliant patients,

found that non-compliant patients

Response: Thanks for the suggestion. We accepted the recommendation and revised the manuscript as shown in red.

6.50 > healed,

delete healed

Response: Thanks for the suggestion. We accepted the recommendation and revised the manuscript as shown in red.

7.55 > quantitative,

maybe qualitative ? It is not clear.

Response: We accepted the recommendation. But we confirm that is “quantitative” here. We mean previous studies on the reasons why patients display poor compliance with GECS are mostly quantitative studies by questionaires.

8.59 > GECS non-compliance were stated,

GECS non-compliance which were stated

Response: Thanks for the suggestion. We accepted the recommendation and revised the manuscript as shown in red.

9.62-64 > are limited. Therefore, researchers should further investigate the reasons for non-compliance with GECS in order to increase patients adherence to the treatment [10],

This sentence is a repetition. Why not to say it in a shorter way, i.e.: "are limited, thus further investigations are needed [10]".

Response: Thanks for the suggestion. We accepted the recommendation and revised the manuscript as shown in red.

10.67 > noncompliance,

non-compliance

Response: Thanks for the suggestion. We accepted the recommendation. But because the sentence where “noncompliance” is used has been deleted, there is no need to modify it.

11.86-87 > After conducting a systematic review of the research,

Omit these words, they are useless

Response: Thanks for the suggestion. We accepted the recommendation and omit these words.

12.104 > Colaizzi's method have,

has

Response: Thanks for the suggestion. We accepted the recommendation and revised the manuscript as shown in the Revised Manuscript with Track Changes.

13.300 > education guidelines,

"education, guidelines". Without comma the sentence is not understandable.

Response: Thanks for the suggestion. We revised this sentences to “In addition, we should reinforce to develop more complete guidelines of health education about the use of GECS based on both medical and practical points of view [5].”

14.319-320 This is a repetition of lines 213-214. Erase the first or the second occurrence.

Response: Thanks for the suggestion. We have erased lines 213-214 and revised it to “which bring difficulties to the patients.”

15.375 > Refernces,

References

Response: Thanks for the suggestion. We accepted the recommendation and revised the manuscript as shown in red.
